# Immunoinformatics Approaches for Vaccine Design: A Fast and Secure Strategy for Successful Vaccine Development

**DOI:** 10.3390/vaccines11020221

**Published:** 2023-01-19

**Authors:** Suraj Singh Rawat, Anand Kumar Keshri, Rimanpreet Kaur, Amit Prasad

**Affiliations:** School of Biosciences and Bioengineering, Indian Institute of Technology Mandi, Kamand 175075, India

Vaccines are major contributors to the cost-effective interventions in major infectious diseases in the global public health space. Vaccine design is a critical and complicated task, where having a successful candidate to induce an effective humoral and cell-mediated immune response against a selected pathogen is ultimately desirable. Notably, throughout the past two centuries, most vaccines were designed using traditional approaches, such as using heat or chemically killed pathogens or by using attenuated pathogens; these approaches almost took approximately 15–20 years to develop into a successful vaccine against any given pathogens due to high rate of failure at advanced stages [1]. In the past few decades, a new area of vaccine design called “Immunoinformatics” has made huge developments and contributed immensely to the area of vaccine design and development [2]. Immunoinformatics, or computational immunology, integrates computational power with the huge amount of genetic and proteomic information collected from pathogens to understand their immune response, and that information is subsequently utilized for the vaccine development [3,4]. Due to recent epidemic outbreaks, such as Zika virus, Influenza virus, SARS and, more recently, the COVID-19 pandemic, the attention of the scientific community has recently been focused toward the unprecedented need for speedy vaccine design. The expansion of the human population and the exploitation of natural habitats of different exotic species has caused humans to interact more with different pathogens. Due to which, emerging infectious diseases are a real threat to humankind. In this scenario, vaccination has proven to be the most effective strategy to controlling the spread of disease and also allows us to study the host–pathogen interactome in detail. As discussed, the traditional approach takes 15–20 years to provide a suitable candidate; however, immunoinformatics-based candidates usually take 2–3 years to develop and also allows us to screen multiple novel candidates simultaneously [5].

Protection against infection can be achieved via two arms of immune response, B-cell-mediated and T-cell-mediated, in which the humoral (B-cell) immune response provides rapid pathogen neutralization or the T-cell-mediated response provides immunological memory to the host, which can be achieved by the integration of dominant epitopes in the vaccine [6]. The steps involved in the immunoinformatics-based multi-epitope vaccine are depicted in Figure 1. In the beginning, linear B-cell epitope prediction tools predicted epitope by analyzing physiochemical properties of different amino acids in sequential manner; however, after the integration of machine learning (ML) algorithms such as support vector machine (SVM), random forest or artificial neural networks (ANN), the comparison of the datasets of different methods to give more optimistic results and avoid misleading predictions was enhanced and the prediction accuracy of the tools was increased to 65.93% [7]. These algorithms operate on pre-defined datasets obtained from the different repositories and cross-validate the sequences to give highly antigenic epitopes. Similarly discontinuous or conformational B-cell epitope prediction tools were also developed to analyze the capability of epitope to acquire different conformations, such as DiscoType, BEpro and Epitopia [8]. Different studies have exploited the predictions of these to analyze the presence of epitopes in the dominant antigens of different pathogens, such as *Acinetobacter baumannii*, bronchitis virus, tuberculosis, *Taenia solium* and *Ascaris lumbricoides* [9,10,11,12,13]. The T-cell epitopes are equally desired for vaccine designing and are predicted by dozens of tools which characterize epitopes based on features, stable amphipathic nature, hydrophobicity and MHC binding efficiency [14]. The latter is critically important, as it provides more specificity and accuracy. Different epitopes induce varied immune responses (Th1 or Th2 response) that are dependent on the inherent nature of the peptide sequence and differentiated using the IEDB database for selection during epitope selection [15]. T-cell epitope prediction can be carried out for MHC-I-specific CD8^+^ cytotoxic T-cells or against the MHC-II-specific helper T-cell population, where CD8^+^ cytotoxic T-cell specific epitope prediction tools utilize machine learning algorithms, such as SVM, ANN and position-specific scoring matrix (PSSM) profile, with varied specificities [16,17]. Similarly, for MHC-II-specific epitopes, SVM, ANN, hidden Markov model and proteochemometrics are based on a quantitative structure activity relationship (QSAR) in order to achieve maximal prediction efficiency [18,19]. The different tools showed varied accuracies and were used according to user demand. Ideal epitopes should be potentially antigenic and should be non-allergic, although it should also possess other parameters such as hydrophobicity and a cationic nature (for efficient deliveries).

The evaluation of critical parameters, such as antigenicity, allergenicity, toxicity, autoimmunity, half-life estimation, solubility and immunogenicity, has been taken into consideration in order to obtain better candidates which can be used for the construction of multiepitope vaccines [20]. Potential epitopes are linked with different linkers to gain a larger sequence carrying both B-cell and T-cell epitopes, which can be used for docking studies. For secondary and tertiary structure predictions, different tools, such as I-Tasser and AlphaFold2.0, have been suggested. Of the two, the latter is the more effective, as it is able to predict structure with more than 90% accuracy compared to the former; however, any structure should pass the Ramachandran plot, so the tools should be used accordingly [21,22,23]. Docking servers perform this task on different algorithms and provide different conformations with docking scores and binding energies, which define the stability of the complexes. The docking of potential vaccine candidates with probable cellular receptors by using different servers, such as HawkDock, HADDOCK 2.0 and Z-DOCK, are required to evaluate the types of responses that can be generated, followed by molecular dynamics studies that shed light on vaccine stability with immune receptors [24,25]. Candidates can be cloned into plasmids of choice, such as pET28a and pET23a, for the bacterial expression and purification along with different affinity tags (like Myc, His, etc.), followed by the removal of endotoxins from affinity purification. Expressed candidates have shown immense potential through immunoinformatics against different pathogens, such as *Escherichia coli*, SARS-CoV-2 and Human Immunodeficiency Virus [26,27].

Although Immunoinformatics has shown immense potential for vaccine development and its potential is now globally recognized, the selection of common epitopes by cross-validation using different tools would enable the global strategic plans to develop efficacious candidates. Compared to the old methods, this new area has made vaccine design more rapid than ever and also exploited certain novel antigenic regions of different proteins which had not been previously investigated. However, this field has several challenges and care needs to be taken regarding its advancement. Since the development of immunoinformatics-based approaches, several tools have come into play which rely on very small datasets, a lack of scheduled maintenance and the quality of predictions, which depend upon background algorithms and may provide false positive sequences; thus, reliability of different prediction tools and databases is another hurdle for vaccine design [28,29]. The major challenge associated with vaccines designed through immunoinformatics is that they need to be validated in in vivo systems, which is a major road bloch at present for this area.

## Figures and Tables

**Figure 1:**
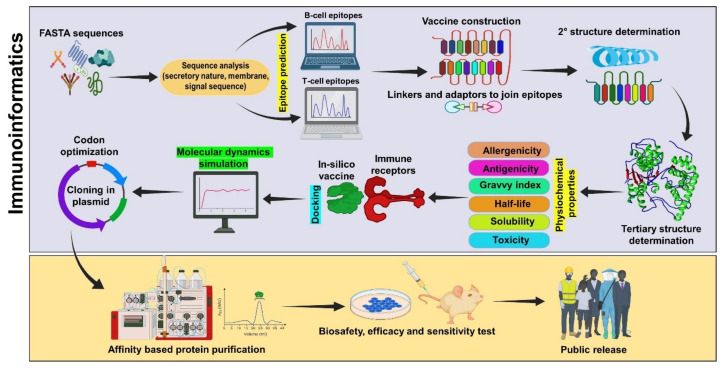
A detailed stepwise pipeline commonly used to construct a multi-epitope peptide vaccine through the immunoinformatics approach.
